# Tumor-Agnostic Therapies in Practice: Challenges, Innovations, and Future Perspectives

**DOI:** 10.3390/cancers17050801

**Published:** 2025-02-26

**Authors:** Sulin Wu, Rajat Thawani

**Affiliations:** Section of Hematology/Oncology, Department of Medicine, University of Chicago, Chicago, IL 60637, USA; sulin.wu@uchicagomedicine.org

**Keywords:** drug development, phase 1 trials, tumor agnostic trials

## Abstract

This review comprehensively analyzes the current landscape of tumor-agnostic therapies in oncology. Tumor-agnostic therapies are designed to target specific molecular alterations rather than the primary site of the tumor, representing a shift in cancer treatment. We discuss recent approvals by regulatory agencies such as the FDA and EMA, highlighting therapies that have demonstrated efficacy across multiple cancer types sharing common alterations. We delve into the trial methodologies that underpin these approvals, emphasizing innovative designs such as basket trials and umbrella trials. These methodologies present unique advantages, including increased efficiency in patient recruitment and the ability to assess drug efficacy in diverse populations rapidly. However, they also entail certain challenges, including the need for robust biomarkers and the complexities of regulatory requirements. Moreover, we examine the promising prospects for developing therapies for rare cancers that exhibit common molecular targets typically associated with more prevalent malignancies. By synthesizing these insights, this review underscores the transformative potential of tumor-agnostic therapies in oncology. It offers a pathway for personalized cancer treatment that transcends conventional histology-based classification.

## Simple Summary

This review explores the emerging field of tumor-agnostic therapies in cancer treatment, which focus on targeting specific molecular changes in tumors rather than their location. This approach marks a significant shift in oncology, as it allows for more personalized treatment options. The review highlights recent approvals by agencies like the FDA and EMA, showcasing therapies that have been effective across various cancer types that share similar molecular features. It also discusses innovative trial designs, such as basket and umbrella trials, which enhance patient recruitment efficiency and quickly evaluate drug effectiveness in diverse patient groups. However, these methods come with challenges, such as the need for strong biomarkers and complex regulatory processes. Additionally, the review emphasizes the potential for developing therapies for rare cancers that have common molecular traits found in more common cancers. Overall, it illustrates the transformative capability of tumor-agnostic therapies, promoting a more personalized approach to cancer treatment that moves beyond traditional methods based on tumor type.

## 1. Introduction

Cancer therapy has undergone a paradigm shift, transitioning from site-specific approaches to molecularly targeted treatments. These treatments focus on shared molecular features rather than the tumor’s anatomical origin. This concept acknowledges cancer as a disease driven by genetic and molecular aberrations, enabling therapies to target universal drivers across diverse tumor types.

The U.S. Food and Drug Administration (FDA) approved pembrolizumab in May 2017, a landmark in oncology [1]. As the first tumor-agnostic therapy, pembrolizumab was approved for adult and pediatric patients with microsatellite instability-high (MSI-H) or mismatch repair-deficient (dMMR) tumors. This milestone validated the feasibility of targeting molecular features irrespective of tumor origin and paved the way for subsequent therapies, such as larotrectinib and entrectinib, which target NTRK fusions [2,3,4,5].

Tumor-agnostic therapies represent a fundamental evolution in oncology, shifting the focus from anatomical classification to molecular drivers. By targeting shared molecular alterations across diverse cancer types, these therapies have achieved remarkable progress. However, challenges remain in designing practical clinical trials, ensuring equitable access, and addressing tumor heterogeneity. This review provides a comprehensive perspective on the current landscape of tumor-agnostic therapies, including (1) detailing the innovative clinical trial designs that enable their development, (2) discussing FDA-approved therapies, and (3) exploring emerging treatments in clinical trials. It also explores barriers to equitable healthcare delivery and proposes strategies to overcome these challenges. By addressing these key themes, this review outlines a roadmap for advancing tumor-agnostic therapies, highlighting their potential to revolutionize cancer care and bridge the gap between scientific innovation and equitable patient access.

As this paradigm continues to reshape oncology, understanding the genetic and molecular landscape of tumors becomes increasingly essential for developing targeted and effective treatments, moving beyond traditional classifications based on the tissue or organ of origin [6,7].

## 2. Benefits of Tumor-Agnostic Therapies

Tumor-agnostic therapies prioritize molecular characteristics, such as mutations, fusions, or biomarker expressions, over tissue-specific attributes. By targeting these molecular alterations, tumor-agnostic therapies enable broader applicability and greater precision.

A key principle in tumor-agnostic strategies is the distinction between driver and passenger mutations in cancer biology, which is central to the rationale for tumor-agnostic therapies [8,9]. Driver mutations are the critical genetic alterations that initiate and sustain tumor growth, making them ideal targets for therapeutic intervention [7]. By targeting these alterations, tumor-agnostic therapies can disrupt the fundamental pathways that underpin cancer cell survival and proliferation [10]. In contrast, while passenger mutations do not directly drive tumorigenesis, they offer valuable insights into the evolution of the tumor and its interaction with the surrounding microenvironment [11,12]. Although these mutations are not primary therapeutic targets, their characterization can enhance the understanding of tumor heterogeneity and resistance mechanisms, further supporting the development of precise, personalized treatment strategies. Therefore, distinguishing between driver and passenger mutations is essential for optimizing tumor-agnostic approaches and improving clinical outcomes.

While the agnostic approach is increasingly applied to common cancers harboring actionable mutations, it is advantageous for rare malignancies with shared molecular alterations across tissue types. For instance, molecular alterations such as NTRK fusions and MSI-H/dMMR status occur across a range of both rare and common tumor types, allowing for broader applications beyond tissue-specific constraints [3,13] and offering effective treatment options for patients who previously had limited or no alternatives. Furthermore, tumor-agnostic strategies are particularly beneficial for cancers unresponsive to conventional treatments predicated on the primary site of the malignancy [14]. By targeting pivotal mutations in cell processes that are fundamental to tumorigenesis, these therapies provide a novel framework for addressing treatment resistance. This methodology is especially pertinent for advanced or metastatic diseases, where the therapeutic objective shifts from focusing on the tumor’s origin to mitigating growth and dissemination. Such an approach expands therapeutic possibilities for patients with otherwise limited options [15].

Additionally, tumor-agnostic therapies also address critical challenges in oncology, particularly for advanced, refractory, rare, and hereditary cancers [3,16,17,18]. These therapies reduce dependency on cytotoxic chemotherapies, minimizing off-target effects and systemic toxicities. By exploiting intrinsic tumor susceptibilities, they achieve higher therapeutic precision and improve the outcomes. For advanced or metastatic cancers with limited site-specific treatment efficacy, tumor-agnostic therapies offer new strategies to mitigate tumor growth and dissemination. Furthermore, these therapies streamline drug development by enabling unified interventions for diverse cancers sharing common genetic drivers, such as BRAF V600E, HER2, NTRK fusions, and ROS1 rearrangements [19,20,21,22,23].

Table 1 summarizes current tumor-agnostic therapies, highlighting their molecular targets and mechanisms of action. This includes immune checkpoint inhibitors, targeted kinase inhibitors, antibody-drug conjugates, and PARP inhibitors. Each therapy type is designed to exploit specific molecular vulnerabilities, reflecting the growing landscape of agnostic treatment options in precision oncology.

The overarching rationale for tumor-agnostic therapies is their ability to bridge molecular precision with broad applicability. By targeting the fundamental driver and the relevant microenvironment of cancer, agnostic therapies align with the principles of precision medicine, offering transformative potential in personalized treatment.

## 3. Trial Designs in Tumor-Agnostic Therapy

The development of tumor-agnostic therapies necessitates innovative clinical trial designs to evaluate their efficacy across diverse patient populations. These therapies target molecular aberrations shared across multiple cancer types, requiring trials that transcend the traditional tumor-specific framework. The following section reviews the significant trial designs used in tumor-agnostic therapy development, emphasizing their unique features and contributions to precision oncology. A table of notable trial designs reviewed below can be found in Table 2. These trials showcase how innovative designs drive tumor-agnostic therapies and precision oncology in the United States, laying the foundation for the future of personalized cancer care.

Master Protocols

Master protocols facilitate the simultaneous evaluation of multiple therapies or hypotheses under a unified framework. These protocols incorporate three primary trial designs—basket trials, umbrella trials, and platform trials. While these designs are not mutually exclusive, they work together to streamline drug development. By leveraging shared infrastructure for tasks such as actionable target selection, data management, and statistical methodologies, these protocols enhance trial efficiency, resource utilization, and patient outcomes. They are particularly advantageous for tumor-agnostic therapies by consolidating operational processes, enhancing statistical rigor, and fostering patient-centered approaches. They boost participant appeal by increasing the likelihood of allocation to experimental arms, while allowing for statistical innovation through shared information between sub-studies. This adaptability enhances the overall efficiency and effectiveness of drug development [24].

Master protocols are especially beneficial for rare cancers or specific cancer subtypes with small patient populations sharing unique molecular profiles. The MOSCATO 01 trial illustrated this potential by assessing the feasibility and clinical benefit of molecular profiling to guide personalized cancer treatment. The trial showed that 7% of successfully screened patients benefited significantly from genomic alteration-driven therapy, underscoring the need for precise patient selection and targeted interventions [25]. By integrating genetic information and disease characteristics, master protocols enhance treatment precision, personalize care, and improve clinical outcomes. Their ability to incorporate multiple hypotheses and therapies within a single framework makes them particularly effective for complex, tumor-agnostic strategies.

However, widespread adoption faces challenges in design, regulation, resources, and cost. These trials require advanced statistical models, robust infrastructure, and sophisticated data management systems. Regulatory complexities necessitate continuous collaboration with authorities to ensure patient safety and compliance. Additionally, extensive and robust biomarker testing with precise cohort selection demands significant investment in sample processing, molecular profiling, and bioinformatics analyses.

The success of master protocols depends on seamless coordination among clinicians, researchers, pharmaceutical companies, and regulatory bodies. Yet, financial barriers related to the costs of high-throughput and comprehensive genomic profiling remain major obstacles to accessibility and scalability [26,27,28,29]. To fully realize the potential of these novel protocols, insurance coverage and official treatment guidelines must evolve in tandem with scientific advancements to promote equitable access. Additionally, integrating these innovative approaches into clinical guidelines will help standardize care, support clinical decision-making, and encourage broader adoption. Without these systemic updates, the benefits of precision oncology may remain inaccessible to many patients, limiting the impact of these groundbreaking strategies.

As we advance master protocols and related trial designs that utilize genomic profiling to establish personalized and adaptive treatment, it is crucial to prioritize education for patients and the public. Enhancing the awareness of the role of genetics and precision medicine in cancer diagnosis and treatment fosters informed decision-making, promotes patient engagement, and builds public trust in innovative cancer therapies. This educational effort is essential for ensuring equitable access and maximizing the impact of precision oncology.

Equally important is educating patients about their rights and protections under laws like the Genetic Information Nondiscrimination Act (GINA), which prohibits discrimination based on genetic information in health insurance and employment [30,31,32]. While GINA primarily focuses on germline genetic information, which protects individuals from discrimination based on inherited genetic risks, it represents a crucial first step in safeguarding participants and sets a model for future insurance coverage and patient rights.

As the integrative analysis of somatic and germline alterations becomes increasingly utilized to better understand cancer development, tumor progression, and treatment resistance, the need for comprehensive education and expanded legal protections grows more critical. These safeguards not only promote broader participation in genomics-driven research but also ensure that patients can pursue advanced testing and targeted treatments without fear of discrimination. By addressing concerns surrounding privacy and genetic discrimination, such legal frameworks strengthen patient engagement and build trust in the evolving landscape of precision oncology.

### 3.1. Basket Trials

Basket trials are a cornerstone of tumor-agnostic therapy development in validating the tumor-agnostic concept and expanding therapeutic options for rare and underserved cancers. They are designed to investigate the efficacy of a single therapeutic agent across cancer types sharing a common molecular feature. These trials shift the focus from traditional anatomical classifications to biomarker-driven enrollment, marking a significant advancement in precision oncology [33,34]. This distinction positions basket trials as a biomarker-driven approach uniquely suited to tumor-agnostic drug development, reflecting the broader shift toward precision medicine. For instance, trials targeting NTRK fusions or MSI-H/dMMR status have demonstrated significant efficacy across various cancers, including thyroid, lung, and colorectal cancers. Notable successes include the approvals of pembrolizumab [18], larotrectinib [35], and entrectinib [36], which underscore the utility of basket trials in advancing tumor-agnostic therapies.

Typically conducted as Phase II open-label studies, basket trials enroll patients based on molecular markers, such as genetic mutations or fusions, regardless of tumor origin. This design allows for including heterogeneous cancer types unified by shared molecular abnormalities under a single protocol. The “basket” metaphor aptly describes this structure, encompassing diverse cancer types into one cohesive study. Endpoints commonly assessed in basket trials include (1) efficacy measures, including the objective response rate (ORR), progression-free survival (PFS), overall survival (OS), and disease control rate (DCR); (2) safety profiles, which evaluate tolerability and adverse events; (3) biomarker efficacy, which assesses the correlation between molecular targets and clinical outcomes; (4) pharmacokinetic (PK) and pharmacodynamic (PD) characteristics, particularly in early phase or expansion cohorts; and (4) patient-reported outcomes, including quality of life (QoL) and symptom burden. They provide a framework to quantify therapeutic benefits, validate novel hypotheses, and bridge the gap between scientific innovation and clinical application, ultimately improving outcomes for diverse patient populations.

Notable examples of the basket trial include the NCI-MATCH (Molecular Analysis for Therapy Choice) trial, which matches patients to therapies based on their tumor’s genomic profile, regardless of cancer type [37,38,39,40]. This trial highlighted the capability of genomic sequencing to identify actionable targets in a substantial proportion of patients, with higher match rates observed in certain rare cancers, reflecting a greater number of actionable mutations. A key strength of the NCI-MATCH trial lies in its platform-like design, which offers exceptional flexibility. The trial allows for the addition or closure of treatment arms without disrupting the core structure, enabling researchers to continuously adapt to emerging scientific discoveries and evolving therapeutic options. Over time, the trial has incorporated updates to its assay platforms and expanded the range of targeted therapies, reflecting its dynamic and adaptable nature. This design not only streamlines drug development but also enhances the trial’s capacity to evaluate multiple hypotheses concurrently, fostering a more efficient path toward precision oncology.

### 3.2. Umbrella Trials

Umbrella trials focus on a single cancer type stratified by distinct molecular or genetic subgroups, enabling the simultaneous evaluation of multiple targeted therapies [41]. This design is particularly suited to tailor treatments based on genetic profiles of different subgroups within the same cancer. For example, in non-small cell lung cancer (NSCLC), patients may be stratified based on biomarkers such as EGFR mutations, ALK rearrangements, or KRAS mutations, with each subgroup receiving a therapy matched to its molecular profile. Adaptive randomization is frequently utilized, allowing dynamic adjustments to trial arms based on interim analyses. This approach ensures the efficient allocation of resources and enhances trial adaptability.

The key advantages of the umbrella trails include comprehensive coverage with efficient resource sharing by enabling the evaluation of multiple therapies across molecularly distinct subtypes of a single cancer in one streamlined protocol. With shared control arms, as recommended by the FDA, resource demands are reduced, and trial efficiency is improved by providing standard treatment to patients without specific markers. By focusing on molecular stratification within a single tumor type, these trials offer valuable insights into cancer heterogeneity and improve the precision of therapeutic interventions. The National Lung Matrix Trial (NLMT) is a prime example, demonstrating the feasibility and utility of umbrella trials in precision oncology [42].

In addition to the common efficacy, pharmacological characteristics, safety profiles, and outcomes as assessed in the basket trial, the umbrella trial focuses on several key endpoints [26]. These include evaluating the tolerability of multiple targeted therapies. For example, the TRIUMPH trial for head and neck squamous cell carcinoma assessed the safety of different targeted agents like alpelisib and poziotinib, ensuring that the toxicity profiles were manageable [43]. Another important aspect of the umbrella trial is to assess the predictive and prognostic value of molecular markers [44]. Finally, the health-related quality of life (HRQoL) will be evaluated to determine the impact of treatments on patient-reported outcomes [45,46].

Umbrella trials are pivotal in precision oncology, balancing distinct risks and significant benefits. By leveraging molecular stratification within a single tumor type, these trials streamline therapeutic interventions with adaptive designs and shared control arms, accelerating drug development and improving patient outcomes. While challenges such as maintaining balanced subgroup sizes, managing overlapping biomarkers, and addressing statistical complexities pose risks of misinterpretation and trial failure [47], particularly for rare molecular subgroups, their role in precision medicine is solidified. A notable example is the NLMT, which demonstrated the feasibility of rapid sequencing for large numbers of tumors, enabling the timely identification of actionable targets for therapy decisions [42].

### 3.3. Platform Trials

Platform trials, or multi-arm, multi-stage (MAMS) trials, are perpetual designs that evaluate multiple therapies simultaneously against a common control group. Due to their adaptability, scalability, and ability to assess combinations of therapies or novel drug candidates in a dynamic framework, these trials are particularly suited for tumor-agnostic therapy development.

Platform trials are characterized by pre-specified adaptation rules that allow discontinuing ineffective interventions based on interim results and adding new therapies as scientific discoveries emerge. Moreover, designing trials to include tests of multiple therapies reduces the time and cost associated with traditional trial designs [48,49,50]. Notably, trials can also be classified as platform trials when adaptive randomization and flexible protocols are employed, allowing the addition of new arms for emerging targets or the discontinuation of ineffective ones [24,51].

Platform trials are uniquely positioned to address the evolving landscape of oncology, where expanding molecular insights continually reveal new actionable targets. Notable examples include the ComboMATCH trial, which explores combination therapies to overcome resistance mechanisms often limiting single-agent efficacy, advancing the boundaries of precision oncology. Different from the NCI-MATCH, the ComboMATCH trial builds on this framework by addressing resistance mechanisms and enhancing therapeutic efficacy through combination therapies [52]. Platform trials represent a critical innovation in precision oncology by offering a flexible and efficient framework for testing multiple therapies against diverse biomarkers. These trials enhance drug development efficiency by accommodating the evolving molecular landscape and expanding therapeutic options for rare and underserved cancer populations. Despite the advantages, the platform trials face significant challenges in implementation due to limited evidence of outcome improvement and ethical concerns. The higher toxicity rates, resource demands, and challenges in ensuring equitable access have raised ethical questions [41,47,53,54,55,56].

### 3.4. N-of-1 Trials

The N-of-1 trial design epitomizes the core principles of personalized medicine, tailoring therapeutic evaluation to an individual patient’s unique molecular and clinical profile [57]. These highly flexible, perpetual trials allow for the simultaneous assessment of multiple therapies, with predefined rules for adding new treatment arms or discontinuing ineffective ones. Such adaptability makes N-of-1 trials particularly relevant for tumor-agnostic therapies, especially in addressing rare cancers or unique molecular profiles [58].

N-of-1 trials leverage comprehensive molecular and clinical data, incorporating genomic, proteomic, and metabolomic analyses to inform therapeutic decisions. Their iterative nature allows real-time treatment optimization, dynamically adapting to patient responses. N-of-1 trials fill critical gaps left by broader clinical studies by evaluating rare biomarkers and tailoring protocols for refractory conditions [59]. Their ability to design bespoke treatment strategies fosters the development of tumor-agnostic and gene-targeted therapies, advancing precision medicine across diverse domains [60,61]. Beyond oncology, N-of-1 trials are increasingly pivotal in genetic diseases and gene therapy, providing a framework for the personalized evaluation of gene-editing technologies. These trials enable the precision targeting of pathogenic mutations while minimizing off-target effects, addressing unique biological challenges in rare genetic disorders [62].

Although formal regulatory approvals based solely on N-of-1 data are extremely rare, these individualized studies have led to groundbreaking therapeutic strategies in ultra-rare diseases. For instance, the custom antisense oligonucleotide milasen was developed for a single patient with Batten disease through an N-of-1 approach, demonstrating how a personalized therapy can emerge from focused, patient-specific investigations [63]. In oncology, while no anticancer drug is currently approved exclusively on N-of-1 evidence, these trials guide compassionate use and off-label prescribing and inform subsequent trial expansions, particularly for patients with unique molecular alterations that lack broader clinical evidence. Consequently, N-of-1 designs can pave the way for more formal, larger-scale investigations, potentially expediting access to highly tailored therapies.

N-of-1 trials are pivotal in evaluating rare biomarkers and tailoring protocols for rare or refractory cancers. They assess therapies for uncommon molecular targets often overlooked in more extensive clinical trials and design bespoke treatment strategies that address these cases’ unique biological and therapeutic challenges. By providing granular insights into the interplay between tumor biology and therapeutic response, N-of-1 trials significantly advance the development of tumor-agnostic therapies, expanding their scope and applicability.

While N-of-1 trials and other innovative designs have significantly advanced tumor-agnostic therapy development, their implementation faces notable challenges related to complexity, resource demands, regulatory oversight, and accessibility [64]. These trials require sophisticated statistical designs and robust infrastructure to manage their intricacies. Although high-throughput molecular profiling and biomarker validation are essential, they demand substantial investments, limiting feasibility in low-sourced settings, despite decreasing costs as these technologies become more integrated into clinical practice. Regulatory challenges further complicate implementation, requiring continuous engagement with authorities to navigate evolving frameworks and ensure compliance. The resource-intensive nature of these trials can also exacerbate disparities in access to cutting-edge therapies.

Despite these hurdles, N-of-1 trials and other adaptive designs continue to lay a solid foundation for precision oncology. By leveraging advanced bioinformatics, real-time analytics, and adaptive methodologies, they further refine the evaluation and implementation of tumor-agnostic therapy, ultimately improving patient outcomes.

## 4. Advancing Precision Oncology: Insights from NCI-MATCH, ComboMATCH, and TAPUR Trials

The NCI-MATCH trial (Molecular Analysis for Therapy Choice, NCT02465060) represents a pioneering benchmark in precision oncology and tumor-agnostic therapies [37,65]. Initiated in 2015 and concluded in 2023, this Phase II basket trial enrolled 1201 patients across 38 arms, with a three-year longitudinal follow-up. Coordinated by the National Cancer Institute (NCI) and the ECOG-ACRIN Research Group, the trial aimed to align patients with specific genetic aberrations to targeted therapies, advancing the understanding of how molecular profiling can guide treatment decisions. The primary endpoint includes the objective tumor response by RECIST criteria, assessing significant tumor shrinkage after treatment. The secondary endpoint is the 6-month progression-free survival (PFS) to evaluate disease stability post-treatment. Benchmark thresholds were stringent: a response rate below 5% was deemed non-promising, while rates between 16 and 25% were considered encouraging [37]. Similarly, a 6-month PFS rate of 35% signified potential therapeutic efficacy. Exclusivity criteria excluded participants previously treated with FDA-approved therapies targeting their identified molecular anomalies, though they could be considered for alternative investigational agents addressing other genetic variations. Overall, the NCI-MATCH trial has successfully demonstrated the feasibility of agnostic therapy by screening a large cohort for genetic anomalies and pairing patients with therapies tailored to their tumors’ molecular profiles.

The ComboMATCH trial (NCT05564377) evolved from the NCI-MATCH framework, addressing its limitations by focusing on combination therapies [66]. While NCI-MATCH prioritized monotherapies, ComboMATCH explores the synergistic potential of targeted therapy combinations (e.g., two targeted agents or targeted therapy with chemotherapy). This design aims to overcome drug resistance and enhance clinical efficacy. This trial has expanded the scope of the MATCH trial to enroll around 2000 patients with the potential for the inclusion of pediatric populations, involving NCI’s National Clinical Trials Network (NCTN) and five U.S. trial groups (e.g., ECOG-ACRIN, SWOG). More specifically, the ComboMATCH trial aims to target the genetic complexity of tumors to establish more durable clinical responses, reflecting a progressive shift in precision medicine, emphasizing the need for personalized strategies that address genetic drivers and resistance mechanisms.

The TAPUR trial (Targeted Agent and Profiling Utilization Registry, NCT02693535) exemplifies a real-world application in agnostic therapy trials [67]. Initiated by the American Society of Clinical Oncology (ASCO) in 2015, TAPUR complements NCI-MATCH by exclusively evaluating the off-label use of FDA-approved therapies for patients with advanced cancers who have exhausted standard treatment options [68]. TAPUR employs a unique design, starting with 10 patients per cohort defined by cancer type, genomic alteration, and therapy. If ≥2 patients respond, enrollment expands to 28. The trial assesses endpoints such as objective tumor response, stable disease at 16 weeks, PFS, OS, and treatment-related adverse events (TrAEs). A key finding was identifying tumor mutational burden (TMB) as a predictive biomarker for pembrolizumab response, influencing its FDA approval for TMB-high cancers [69]. Overall, TAPUR bridges the gap between clinical trials and real-world applications, offering valuable insights into the efficacy and safety of targeted therapies in diverse patient populations.

The insights from the TAPUR trial, alongside findings from NCI-MATCH, have significantly advanced the field of targeted cancer treatment. These studies validate the potential of precision medicine, demonstrating that genetically informed therapies can achieve more effective and personalized care. NCI-MATCH highlighted the critical role of molecular profiling in aligning therapies with genetic aberrations, irrespective of cancer type, while providing valuable insights into the efficacy of targeted agents and the mechanisms driving treatment resistance. Building on this foundation, ComboMATCH explored combination therapies to overcome resistance and improve clinical outcomes. Simultaneously, the ASCO-sponsored TAPUR trial offered real-world evidence on applying FDA-approved targeted therapies for off-label indications, enriching the knowledge base on their performance across diverse cancer types and genetic profiles [70].

These trials underscore the transformative potential of genomic profiling and the precise application of targeted therapies, paving the way for innovative treatment paradigms tailored to the unique genetic landscapes of individual tumors and rare cancer subtypes. Together, they demonstrate the continued progress of oncology toward more nuanced and effective therapeutic strategies.

## 5. Approvals of Tumor-Agnostic Therapies

Tumor-agnostic therapies have revolutionized cancer treatment by focusing on tumors’ molecular and genetic features rather than their location in the body. These therapies signify a significant milestone in precision oncology, addressing unmet needs in diverse patient populations, particularly those with rare or treatment-resistant cancers. As of 2024, several tumor-agnostic therapies have received regulatory approval from the U.S. Food and Drug Administration (FDA) and the European Medicines Agency (EMA), underscoring their growing clinical significance (Table 3).

Regulatory agencies like the FDA and EMA have implemented expedited review pathways, including the Breakthrough Therapy Designation and Priority Review, to accelerate the development and approval of tumor-agnostic therapies. These frameworks provide clear guidance on clinical trial design, biomarker validation, and regulatory compliance, ensuring a streamlined process for bringing these transformative treatments to market.

### 5.1. FDA-Approved Tumor-Agnostic Therapies

The approval of pembrolizumab (Keytruda) in 2017 marked the beginning of the tumor-agnostic therapy era. This PD-1 inhibitor was approved for adult and pediatric patients with unresectable or MSI-high (MSI-H) or mismatch repair-deficient (dMMR) solid tumors that had progressed after prior treatment. This landmark approval demonstrated the feasibility and efficacy of targeting genetic biomarkers over the tumor origin, setting a precedent for subsequent research [1].

Following pembrolizumab, larotrectinib (Vitrakvi) was approved in 2018 for its efficacy against tumors with neurotrophic receptor tyrosine kinase (NTRK) gene fusions [5]. Clinical trials revealed its remarkable efficacy across multiple tumor types, emphasizing the potential of tumor-agnostic therapies to provide effective options for patients with rare genetic mutations.

In 2019, entrectinib (Rozlytrek) joined the list of tumor-agnostic therapies approved for NTRK fusion-positive tumors and ROS1-positive non-small cell lung cancer (NSCLC), including those with central nervous system metastases [4]. Its ability to prolong progression-free survival, even in patients with advanced disease, has positioned it as a vital tool in precision oncology.

Building on these advancements, Dostarlimab (Jemperli) received FDA approval in 2021 for patients with advanced or recurrent cancers exhibiting dMMR [71,72,73]. Its efficacy across diverse tumor types highlights the potential of these therapies to transform cancer care by targeting shared molecular vulnerabilities.

In 2022, the FDA approved the combination of Dabrafenib (Tafinlar) and Trametinib (Mekinist) for cancers with the BRAF V600E mutation, marking a pivotal advancement in precision oncology [74]. This decision, grounded in robust evidence from the NCI-MATCH and ROAR trials [75,76], highlighted the therapeutic potential of targeting complex molecular drivers across multiple tumor types, including ovarian and central nervous system cancers. Additionally, the combination of Dabrafenib and Trametinib with immunotherapy demonstrated enhanced efficacy, yielding promising response rates and durable outcomes, further underscoring the potential of integrative therapeutic strategies in addressing challenging oncogenic mutations [77].

Most recently, in 2024, fam-trastuzumab deruxtecan-nxki (Enhertu) was approved as the first tumor-agnostic HER2-directed therapy for patients with metastatic HER2-positive solid tumors [78]. This innovative antibody-drug conjugate underscores the potential of tumor-agnostic approaches to redefine treatment paradigms for biomarker-driven cancers.

### 5.2. EMA-Approved Tumor-Agnostic Therapies

The EMA has also recognized the value of tumor-agnostic therapies, granting approvals to treatments that align with FDA indications [79]. These include pembrolizumab for MSI-H and TMB-H tumors, larotrectinib and entrectinib for NTRK fusion-positive tumors, and Dabrafenib with Trametinib for BRAF V600E-mutated cancers. However, it is important to note that, as of February 2025, the European Medicines Agency (EMA) has not granted a similar tumor-agnostic approval for Enhertu. In the European Union, Enhertu’s approvals remain specific to certain cancer types, such as breast cancer (HER2-positive and HER2-low), gastric or gastroesophageal junction adenocarcinoma (HER2 -positive), and NSCLC with activating HER2.

The therapies highlighted in Table 3 exemplify the profound impact of tumor-agnostic approaches on oncology. By targeting the molecular drivers of cancer, these treatments transcend the traditional focus on tumor origin, offering personalized and effective solutions for diverse patient populations. This shift represents a critical evolution in cancer care, paving the way for innovative strategies prioritizing patient-specific genetic landscapes over conventional classifications. Tumor-agnostic therapies continue to redefine the possibilities of precision medicine, heralding a new era of tailored and impactful cancer treatments.

## 6. Future of Tumor-Agnostic Therapy Development

Identifying the genetic landscape across malignancies is foundational to developing tumor-agnostic therapies. These strategies leverage universal biomarkers and associated pathways to address cancer progression, metastasis, and resistance, representing a paradigm shift in oncology. Built on a robust foundation of genomic technologies, multiomic integration, computational modeling, and AI-driven analytics, this innovation has enabled the identification of molecular drivers and functional biomarkers critical to tumorigenesis. These advancements guide the development of therapies that transcend tumor location and prioritize molecular precision.

The synthesis of genomic, transcriptomic, proteomic, metabolomic, histopathological, and radiographic data has become fundamental in identifying actionable targets. Integrating these diverse datasets exposes common molecular signatures across tumor types and refines our grasp of the functional roles of oncogenic mutations. This holistic approach allows for precise patient stratification and the identification of biomarkers with predictive and prognostic value for monitoring disease progression and evaluating therapeutic responses [80,81,82,83].

Large-scale initiatives like The Cancer Genome Atlas (TCGA) and the International Cancer Genome Consortium (ICGC) have advanced biomarker discovery [84]. Since its inception, TCGA has provided a comprehensive database of cancer genomic profiles, aiding in identifying treatment-response biomarkers and elucidating mechanisms underlying drug resistance. Similarly, ICGC focuses on genomic, epigenomic, and transcriptomic analyses of over 50 cancer types, facilitating the discovery of universal biomarkers critical to the success of tumor-agnostic therapies. By correlating large datasets with clinical outcomes, these consortia refine diagnostic frameworks and guide novel therapies, especially for rare cancers with limited treatment options.

Artificial Intelligence (AI) and Machine Learning (ML) have become integral in managing extensive genomic and clinical datasets, driving precision in biomarker discovery and clinical trial design. These technologies facilitate the identification of complex patterns within high-dimensional datasets, which can be difficult to discern using traditional analytical methods [85,86,87]. AI-driven pattern recognition and predictive modeling—including both language-based and image-based approaches—greatly enhance biomarker discovery and patient stratification. Natural Language Processing (NLP) is particularly useful for analyzing unstructured text from lab reports, clinical notes, and trial data, allowing for the extraction of valuable insights regarding biomarker expressions, mutations, and treatment responses [88,89,90,91]. This capability supports integrating diverse clinical data, leading to more informed decision-making. On the other hand, image-based analysis in radiogenomics and pathology employs deep learning techniques, such as Convolutional Neural Networks (CNNs) and Recurrent Neural Networks (RNNs), helping to identify patterns and features not identifiable with conventional approaches [92,93,94,95].

Reinforcement learning (RL), integrating language-based and image-based AI, enables comprehensive biomarker identification, combining textual insights with visual data analysis. This dual approach uncovers correlations that have been previously unrecognized, expanding the scope of tumor-agnostic therapies. The RL algorithm further optimizes treatment strategies and adaptive clinical trials, while bioinformatics pipelines like the Genome Analysis Toolkit (GATK) and OpenCRAVAT streamline variant annotation and interpretation. Software platforms such as TensorFlow, PyTorch, and scikit-learn support ML model development, while IBM Watson for Oncology and Google’s DeepVariant specialize in genomic data analysis [96,97]. Integrating AI and ML into agnostic cancer clinical trials enhances precision, scalability, and efficiency. These technologies not only accelerate biomarker identification and validation but also improve patient selection and treatment optimization, ultimately advancing personalized oncology care.

Innovative tools for biomarker discovery further complement these computational approaches. Protein modeling such as AlphaFold and Missense3D provide estimated structures of oncogenic proteins, enabling the identification of druggable binding sites to inform the design of targeted therapies [98,99]. CRISPR-based screening systematically identifies essential genes for tumor survival, precisely targeting molecular vulnerabilities. Furthermore, clinical phenotyping enriches molecular findings with real-world data on treatment responses and resistance patterns, enabling the design of therapies optimized for dynamic tumor evolution. Furthermore, image-based diagnostics, such as radiomics and pathomics, further enhance tumor-agnostic strategies by correlating imaging features with molecular drivers and therapeutic outcomes. By integrating imaging biomarkers with genomic data, researchers refine prognostic assessments and identify surrogate markers for evaluating the efficacy of therapies.

This multidisciplinary approach, diverging from traditional empirical methods, has made tumor-agnostic therapies more precise and adaptable. It opens new avenues for drug development, repurposing existing treatments, and addressing the complex molecular mechanisms of cancer progression. This heralds a future where cancer care is tailored to the unique genetic landscapes of individual tumors, delivering transformative benefits to patients across diverse cancer types.

## 7. Challenges in Biomarker Development

Biomarkers are central to tumor-agnostic therapies, which target molecular alterations regardless of tumor origin. They identify key pathways driving cancer growth and streamline patient selection, ensuring treatments are matched to those most likely to benefit. Biomarkers also enable the ongoing monitoring of therapeutic effectiveness. However, implementing tumor-agnostic approaches faces significant hurdles, including complex biomarker validation across diverse malignancies and the challenge of enrolling suitable patient cohorts into clinical trials.

A primary challenge in deploying tumor-agnostic therapies is the genomic diversity and mutation load within tumors [100,101]. The cancer heterogeneity across different tissues and within individual tumors poses substantial challenges for developing tissue-agnostic biomarkers. Even if a molecular target appears in multiple malignancies, the effectiveness of a given therapy can vary drastically due to co-occurring mutations, distinct microenvironments, and therapy-induced resistance. Biomarkers such as TMB or MSI also have varying clinical relevance across tumor subtypes, complicating standardization efforts. A clear illustration of these nuances is the variable efficacy of sotorasib (targeting KRAS^G12C^) in NSCLC versus colorectal cancer (CRC), where a combination with panitumumab is needed to match the results observed in NSCLC [102]. Likewise, osimertinib, with significant efficacy in treating NSCLC with the EGFR T790M mutation, shows less consistent outcomes in other tumor contexts, underscoring the need to address co-occurring alterations and heterogeneous tumor ecosystems [103]. Moving forward, comprehensive molecular profiling can reveal emerging subclones that may drive resistance, guiding adaptive treatment strategies. Developers must also explore synergistic pathways and validate biomarkers in models that accurately represent each tumor type. Finally, adaptive clinical trial designs, with stratified patient cohorts and real-time biomarker assessments, are crucial to detect early signals of differential efficacy and to incorporate combination regimens when monotherapy proves insufficient.

Current tumor-agnostic therapies predominantly target a narrow range of biomarkers associated with specific mutations or genomic instability, such as NTRK fusions, BRAF V600E, MSI-H, or TMB. This focus excludes many patients whose tumors might benefit from agnostic therapies targeting alternative mechanisms, like epigenetic modifications or non-coding RNA dysregulation. Expanding the biomarker repertoire is crucial to widen the impact and reach of tumor-agnostic therapies.

In clinical practice, the complexity of biomarker interpretation is heightened when patients exhibit multiple mutations or overlapping genetic signatures. Deciding how to prioritize treatment for patients with various actionable biomarkers, such as MSI-H combined with a BRAF V600E mutation, presents a clinical dilemma. Moreover, integrating biomarker data with other patient-specific factors, such as comorbidities or prior treatment history, requires sophisticated decision-making frameworks that are not yet widely available. Meanwhile, tumors frequently acquire additional mutations or develop resistance mechanisms over time, underscoring the need for adaptive treatment strategies and novel trial endpoints in future studies, such as molecular remission.

The robust and comprehensive assessment of biomarkers and corresponding molecular landscape often requires advanced genomic methods such as next-generation sequencing (NGS), which can pose substantial financial hurdles for both patients and healthcare systems. In low-resource settings, these challenges are compounded by inadequate infrastructure and limited reimbursement, restricting patient eligibility for tumor-agnostic therapies and exacerbating care disparities [104,105]. Even where testing is available, technical limitations that lead to false negatives may exclude appropriate patients from effective treatments. Moreover, the absence of universally accepted guidelines on integrating biomarker testing into routine clinical workflows complicates real-world implementation even further.

To address these issues, developing and validating biomarkers for agnostic therapies require clinical evidence often generated through large-scale trials [79,106]. The rarity of some biomarkers, such as NTRK fusions or rare BRAF mutations, poses challenges in assembling adequate trial cohorts. The lack of standardized definitions and thresholds for biomarkers like TMB also leads to variability in clinical decision-making and outcomes. The evolving regulatory guidelines for biomarker validation and approval add to the uncertainty, complicating the landscape for developers and clinicians.

Furthermore, the high cost of biomarker testing and agnostic therapies poses significant financial barriers for patients and healthcare systems [107]. Regulatory bodies demand extensive data to approve new biomarkers, yet the rarity of specific mutations complicates the generation of robust evidence. These economic and regulatory challenges decelerate the adoption of biomarkers and the rollout of tumor-agnostic therapies, calling for innovative solutions to bridge these gaps and enhance cancer care delivery.

In summary, while tumor-agnostic therapies have transformative potential in precision oncology, overcoming the challenges of biomarker heterogeneity, limited testing accessibility, evolving regulatory requirements, and trial recruitment are all critical. Addressing these issues will require innovative clinical trial designs, adaptive regulatory frameworks, and the broader availability of genomic technologies to fully harness the promise of tumor-agnostic treatments.

## 8. Future Perspectives

The future of tumor-agnostic therapies in cancer treatment is poised for significant expansion and refinement as precision medicine advances. By targeting molecular alterations rather than tumor types, these approaches offer a universal framework for therapy, transforming how we understand and manage cancer.

As genomic sequencing technologies grow more sophisticated and accessible, new biomarkers with agnostic potential will increasingly emerge. This expansion should widen the range of treatable cancers and spur rigorous efforts to validate biomarkers’ predictive value in diverse patient populations. Concurrently, innovative molecular profiling methods, including single-cell sequencing and spatial omics, will deepen our understanding of tumor behavior on an individualized basis, enabling the development of more potent therapies for primary, metastatic, and recurrent disease [108,109,110,111].

AI and ML play a central role in synthesizing large-scale genomic data, identifying previously unrecognized patterns, and speeding the stratification of tumor subgroups most likely to benefit from agnostic therapies. These insights guide patient selection and inform combination regimens that target or delay therapeutic resistance.

Beyond small molecules and antibody-based treatments, cellular therapies are evolving in tandem with tumor-agnostic approaches [112,113]. T-cell engineering and CAR T-cell strategies have traditionally focused on disease-specific targets (e.g., CD19 in B-cell malignancies) [20]. However, harnessing universal biomarkers and engineered receptors may eventually enable these immunotherapies to adopt a more agnostic framework. A promising frontier in this evolution is the use of Tumor-Infiltrating Lymphocytes (TILs) [114,115,116]. Their ability to recognize a wide array of tumor neoantigens makes them uniquely positioned for tumor-agnostic applications. Emerging strategies involve isolating and expanding TILs from various solid tumors, regardless of tissue origin, and reintroducing them to patients to mount a robust anti-tumor response. Early clinical studies have shown encouraging results, particularly in tumors with high mutational burden or rich immune infiltration [117,118,119,120]. Additionally, antibody-drug conjugates (ADCs) further illustrate the potential for designing immunotherapies around shared tumor targets, bridging the gap between site-specific and agnostic paradigms [121,122,123]. As cellular and antibody-based therapies advance and integrate with genomic profiling and biomarker-driven selection, they hold the potential to become a cornerstone of tumor-agnostic immunotherapy, offering new treatment avenues for patients with otherwise limited options.

As research clarifies tumor genetics and microenvironments, treatment resistance and tolerance remain prime obstacles. Novel strategies, including sequential or combination regimens of multimodality treatments, may be essential to achieving durable responses. Reflecting these advances, regulatory bodies have begun revising approval pathways for agnostic therapies, supporting more rapid bench-to-bedside transitions. For instance, the FDA continues to refine guidelines around biomarker testing, companion diagnostics, and accelerated approvals, helping patients with targetable genetic alterations access emerging treatments sooner.

## 9. Conclusions

In summary, the future for tumor-agnostic therapies is bright. As new biomarkers emerge, molecular profiling matures, and cutting-edge modalities like AI-driven analytics and cellular immunotherapy gain traction, these strategies will increasingly shape cancer care—offering more personalized and effective treatments across a growing spectrum of tumor types worldwide.

## Figures and Tables

**Table 1:** Agnostic therapies in cancer. This table provides an overview of the different therapy types classified as tumor-agnostic treatments in cancer. It summarizes the key therapies currently available or under investigation, their notable examples, molecular targets, and mechanisms of action. The therapies listed include immune checkpoint inhibitors, targeted kinase inhibitors, antibody-drug conjugates (ADCs), PARP inhibitors, oncolytic viruses, RNA-targeted therapies, synthetic lethality-based agents, tumor vaccines, and radioligand therapies. Each category highlights the innovative strategies employed to address shared molecular drivers across various tumor types, independent of their anatomical origins. The table underscores the diversity and complexity of tumor-agnostic approaches, reflecting their transformative potential in precision oncology.

Therapy Type	Mechanism of Action	Examples	Targets
Immune Checkpoint Inhibitors (ICIs)	Enhances immune recognition and response against tumors	Pembrolizumab, Dostarlimab	MSI-H/dMMR, TMB
Targeted Kinase Inhibitors	Inhibits aberrant signaling pathways driving tumor growth	Larotrectinib, entrectinib, Dabrafenib/Trametinib	NTRK Fusions, ROS1 Rearrangements, BRAF V600E
Antibody-Drug Conjugates (ADCs)	Delivers cytotoxic agents specifically to cancer cells	Trastuzumab Deruxtecan (Enhertu), Sacituzumab Govitecan	HER2, TROP2
PARP Inhibitors	Exploits DNA repair vulnerabilities in cancer cells	Olaparib, Rucaparib	Homologous Recombination Deficiencies (e.g., BRCA mutations)
Oncolytic Viruses	Selectively infects and lyses cancer cells	Talimogene Laherparepvec (T-VEC)	Tumor-Specific Antigens
RNA-Targeted Therapies	Disrupts transcription or translation of oncogenes	siRNA, Antisense Oligonucleotides	Oncogenic Transcripts
Synthetic Lethality-Based Agents	Targets vulnerabilities due to genetic defects	ATR Inhibitors	DNA Damage Response Pathways
Tumor Vaccines	Stimulates immune response against tumor-specific antigens	Neoantigen Vaccines	Shared Neoantigens
Radioligand Therapies	Combines radioactive isotopes with ligands targeting tumors	177Lu-FAP-2286 FAP-23	Tumor Markers (e.g., Fibroblast Activation Protein (FAP))

**Table 2:** Notable clinical trials in tumor-agnostic therapy. This table summarizes notable clinical trials in tumor-agnostic therapy, showcasing the evolution of innovative trial designs, therapeutic targets, and patient outcomes. The trials listed, including basket, umbrella, platform, and N-of-1 designs, reflect the diversity in methodologies used to evaluate tumor-agnostic therapies. Each trial utilizes genomic profiling to guide patient selection, ensuring therapeutic interventions are matched to specific molecular targets or abnormalities identified through comprehensive genomic analysis. Key features such as trial objectives, patient cohorts, therapeutic interventions, and significant findings are highlighted, offering insights into the impact of these trials on advancing precision oncology and shaping the future of cancer treatment. This approach underscores the central role of personalized medicine in the current landscape of cancer therapy, focusing on genomic-driven strategies to optimize patient care.

Trial Type	Trial Name	Purpose	Highlights
Basket	NCI-MATCH Trial	Matched patients to therapies based on actionable molecular targets.	Demonstrated feasibility of genomic sequencing for diverse cancers.
	KEYNOTE-158	Evaluated pembrolizumab in MSI-H or dMMR tumors.	Supported pembrolizumab approval as tumor-agnostic therapy.
	Vitrakvi Basket Trials	Assessed larotrectinib for NTRK fusions.	Efficacy demonstrated across multiple cancer types; FDA approval.
	ROZLYTREK Basket Trial	Evaluated entrectinib for ROS1-positive or NTRK fusion-positive cancers.	Resulted in FDA approval for tumor-agnostic indications.
Umbrella	National Lung Matrix Trial (NLMT)	Stratified NSCLC patients by biomarkers to evaluate targeted therapies.	Showed feasibility of rapid sequencing for therapy decisions.
	A Lung-MAP Study	Tested therapies for advanced NSCLC with specific genomic alterations.	Focused on biomarker-driven therapies within NSCLC cohorts.
	ALCHEMIST Trial	Tested adjuvant therapies for resected NSCLC based on biomarkers.	Refined precision therapy for early-stage NSCLC.
	BATTLE-2	Investigated biomarker-directed targeted therapies in NSCLC.	An early adaptive biomarker-driven trial design.
Platform	ComboMATCH Trial	Focused on combination therapies to overcome resistance mechanisms.	Built on NCI-MATCH to test combination strategies.
	I-SPY 2 Trial	Evaluated novel therapies for high-risk breast cancer.	Adaptive and perpetual design for breast cancer therapies.
	GBM AGILE	Studied therapies for glioblastoma using a platform approach.	Integrated multiple experimental arms with a shared control.
N-of-1	Pediatric MATCH	Matched children with advanced cancers to targeted therapies.	Adapted MATCH design for pediatric oncology.
	Individualized Cancer Therapy (iCat) Study	Analyzed personalized therapeutic recommendations based on genomic profiling.	Focused on individual genomic-driven therapy.
	Rare Tumor Precision Oncology Program (RTPOP)	Focused on rare tumors with unique molecular features.	Addressed gaps in rare malignancies with N-of-1 design.
	The TAPUR Study	Evaluated FDA-approved drugs for off-label use based on tumor profiling.	Pragmatic approach to N-of-1 trials in precision oncology.

**Table 3:** FDA and EMA-approved tumor-agnostic therapies. This table provides an overview of tumor-agnostic therapies approved by the U.S. Food and Drug Administration (FDA) and the European Medicines Agency (EMA). The therapies listed target molecular drivers such as NTRK fusions, microsatellite instability-high (MSI-H), mismatch repair-deficient (dMMR), TMB, and BRAF mutations across various tumor types, irrespective of their anatomical origin. Key information includes the therapy name, target biomarker, approval year, clinical trial evidence, and specific indications. This table highlights the pivotal role of these therapies in advancing precision oncology and their transformative impact on cancer treatment paradigms.

Therapy	Indication by FDA	Indication by EMA	Mechanisms of Actions
Pembrolizumab (Keytruda)	Adult and pediatric patients with unresectable or metastatic, MSI-H or dMMR solid tumors that have progressed following prior treatment and who have no satisfactory alternative treatment options.	adult and pediatric patients with unresectable or metastatic MSI-H or dMMR solid tumors that have progressed following prior therapy and when no satisfactory alternative treatment options exist.	Monoclonal antibody targeting PD-1 receptor, enhancing T-cell-mediated immune response against tumor cells.
Larotrectinib (Vitrakvi)	Adult and pediatric patients with solid tumors harboring an NTRK gene fusion without a known acquired resistance mutation, that are metastatic or where surgical resection is likely to result in severe morbidity, and where patients have no satisfactory alternative treatments or have progressed following treatment.	Adult and pediatric patients with solid tumors that display an NTRK gene fusion, who have a disease that is locally advanced, metastatic, or where surgical resection is likely to result in severe morbidity, and who have no satisfactory treatment options.	Selective inhibitor of tropomyosin receptor kinases (TRK) A, B, and C, leading to inhibition of tumor growth driven by NTRK gene fusions.
Entrectinib (Rozlytrek)	Adult and pediatric patients 12 years of age and older with solid tumors that have an NTRK gene fusion without a known acquired resistance mutation, are metastatic or where surgical resection is likely to result in severe morbidity and have no satisfactory alternative treatments or have progressed following treatment.	Adult and pediatric patients 12 years and older with solid tumors expressing an NTRK gene fusion, who have a disease that is locally advanced, metastatic, or where surgical resection is likely to result in severe morbidity, and who have no satisfactory treatment options.	Inhibitor for TRK A/B/C, ROS1, and ALK tyrosine kinases, blocking downstream signaling pathways involved in tumor cell proliferation and survival.
Dostarlimab (Jemperli)	Adult patients with dMMR recurrent or advanced endometrial cancer that has progressed on or following prior treatment with a platinum-containing regimen.	Adult patients with dMMR/MSI-H recurrent or advanced endometrial cancer that has progressed on or following prior platinum-based chemotherapy.	Monoclonal antibody that binds to PD-1 receptor, blocking its interaction with PD-L1 and PD-L2, thereby enhancing T-cell-mediated immune responses against tumor cells.
Dabrafenib (Tafinlar) + Trametinib (Mekinist)	Adult and pediatric patients aged 6 years and older with unresectable or metastatic solid tumors with BRAF V600E mutation who have progressed following prior treatment and have no satisfactory alternative treatment options.	Adult patients with unresectable or metastatic solid tumors with a BRAF V600 mutation, where no satisfactory treatment options are available.	Dabrafenib inhibits mutant BRAF V600E kinase activity. Trametinib inhibits MEK1 and MEK2 activation and kinase activity. Combined, they block the MAPK/ERK signaling pathway, reducing tumor cell proliferation.
Selpercatinib (Retevmo)	Adult patients with locally advanced or metastatic solid tumors with a RET gene fusion that have progressed on or following prior systemic treatment or who have no satisfactory alternative treatment options.	Adult patients with advanced RET fusion-positive thyroid cancer who require systemic therapy following prior treatment with sorafenib and/or lenvatinib.	Selective RET kinase inhibitor, blocking RET-dependent signaling and proliferation in tumor cells with RET gene fusions.
Fam-trastuzumab deruxtecan-nxki (Enhertu)	Adult patients with unresectable or metastatic HER2-positive solid tumor	Adult patients with unresectable or metastatic HER2-positive breast, gastric, GE junction and non-small cell lung cancer	Antibody-drug conjugate combining trastuzumab (anti-HER2 antibody) with a topoisomerase I inhibitor, delivering cytotoxic chemotherapy directly to HER2-expressing tumor cells.
